# *N*-(2-Nitro­phenyl­carbamothio­yl)acetamide

**DOI:** 10.1107/S1600536812016947

**Published:** 2012-04-28

**Authors:** Durre Shahwar, M. Nawaz Tahir, Muhammad Mansha Chohan, Muhammad Akmal Khan, Nadeem Ahmad

**Affiliations:** a Department of Chemistry, Government College University, Lahore, Pakistan; b Department of Physics, University of Sargodha, Sargodha, Pakistan; c Department of Chemistry, University of Engineering and Technology, Lahore 54000, Pakistan

## Abstract

In the title compound, C_9_H_9_N_3_O_3_S, the benzene ring and the *N*-carbamothio­ylacetamide unit are oriented at a dihedral angle of 54.82 (4)°. The dihedral angle between the ring and its attached nitro group is 28.54 (12)°. An intra­molecular, bifurcated N—H⋯(O,O) hydrogen bond generates two *S*(6) rings. In the crystal, inversion dimers linked by pairs of N—H⋯S hydrogen bonds generate *R*_2_^2^(8) loops. Weak C—H⋯O inter­actions link the dimers.

## Related literature

For related structures, see: Shahwar *et al.* (2012**a* ▶,b* ▶,*c* ▶). For graph–set notation, see: Bernstein *et al.* (1995 ▶).
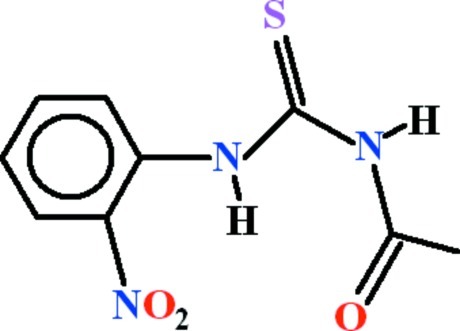


## Experimental

### 

#### Crystal data

C_9_H_9_N_3_O_3_S
*M* *_r_* = 239.25
Monoclinic, 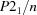

*a* = 4.1992 (1) Å
*b* = 11.6081 (3) Å
*c* = 22.1035 (6) Å
β = 94.815 (1)°
*V* = 1073.63 (5) Å^3^
*Z* = 4
Mo *K*α radiation
μ = 0.30 mm^−1^
*T* = 296 K
0.35 × 0.15 × 0.13 mm

#### Data collection

Bruker Kappa APEXII CCD diffractometer
Absorption correction: multi-scan (*SADABS*; Bruker, 2005 ▶) *T*_min_ = 0.945, *T*_max_ = 0.965
10065 measured reflections
2711 independent reflections
1969 reflections with *I* > 2σ(*I*)
*R*_int_ = 0.029

#### Refinement

*R*[*F* ^2^ > 2σ(*F* ^2^)] = 0.039
*wR*(*F* ^2^) = 0.105
*S* = 1.01
2711 reflections
146 parameters
H-atom parameters constrained
Δρ_max_ = 0.22 e Å^−3^
Δρ_min_ = −0.22 e Å^−3^

### 

Data collection: *APEX2* (Bruker, 2009 ▶); cell refinement: *SAINT* (Bruker, 2009 ▶); data reduction: *SAINT*; program(s) used to solve structure: *SHELXS97* (Sheldrick, 2008 ▶); program(s) used to refine structure: *SHELXL97* (Sheldrick, 2008 ▶); molecular graphics: *ORTEP-3 for Windows* (Farrugia, 1997 ▶) and *PLATON* (Spek, 2009 ▶); software used to prepare material for publication: *WinGX* (Farrugia, 1999 ▶) and *PLATON*.

## Supplementary Material

Crystal structure: contains datablock(s) global, I. DOI: 10.1107/S1600536812016947/hb6744sup1.cif

Structure factors: contains datablock(s) I. DOI: 10.1107/S1600536812016947/hb6744Isup2.hkl

Supplementary material file. DOI: 10.1107/S1600536812016947/hb6744Isup3.cml

Additional supplementary materials:  crystallographic information; 3D view; checkCIF report

## Figures and Tables

**Table 1:** Hydrogen-bond geometry (Å, °)

*D*—H⋯*A*	*D*—H	H⋯*A*	*D*⋯*A*	*D*—H⋯*A*
N2—H2⋯O1	0.86	2.23	2.661 (2)	111
N2—H2⋯O3	0.86	1.93	2.630 (2)	137
N3—H3*A*⋯S1^i^	0.86	2.59	3.4371 (14)	168
C9—H9*C*⋯O2^ii^	0.96	2.51	3.428 (3)	161

Symmetry codes: (i) 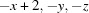; (ii) 
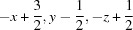
.

**Fig. 1.**
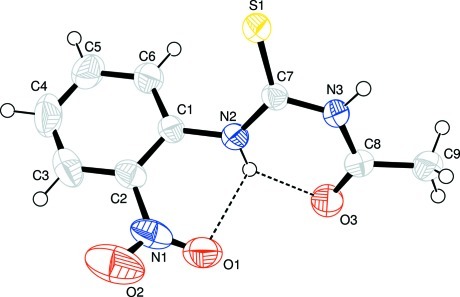
View of the title compound with displacement ellipsoids drawn at the 50% probability level. The dotted lines represent the intra-molecular H-bondings.

**Fig. 2.**
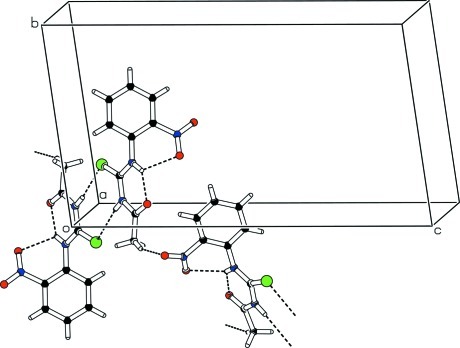
Partial packing diagram showing inversion dimers linked by pairs of N—H···S hydrogen bonds with R22(8) ring motifs.

## supplementary crystallographic information

### Comment

The title compound I (Fig. 1) is in continuation to synthesize different derivatives of *N*-carbamothioylacetamide.

We have reported the crystal structures of *N*-(2-methylphenylcarbamothioyl)acetamide (Shahwar *et al.*, 2012*a*), *N*-(3-chlorophenylcarbamothioyl)acetamide (Shahwar *et al.*, 2012*b*) and *N*-(phenylcarbamothioyl)acetamide (Shahwar *et al.*, 2012*c*) which are related to the (I).

In (I), the phenyl ring A (C1–C6) and the *N*-carbamothioylacetamide moiety B (N2/C7/S1/N3/C8/O3/C9) are planar with r. m. s. deviation of 0.0028 Å and 0.0181 Å, respectively. The dihedral angle between A/B is 54.82 (4)°. The nitro group C (O1/N1/O2) is of course planar. The dihedral angle between A/C and B/C are 28.68 (18) and 66.59 (12)°, respectively. There exist intramolecular H–bonding of N—H···O type (Table 1, Fig. 1) with two *S*(6) ring motifs (Bernstein *et al.*, 1995). The molecules are dimerized due to N—H···S type of hydrogen bonds with *R*_2_^2^(8) ring motifs (Table 1, Fig. 2). The dimers are interlinked from CH_3_ groups due to C—H···O H–bondings (Table 1, Fig. 2) with nitro groups.

### Experimental

Acetylchloride (0.1 mol, 7.13 ml) was added dropwise to a stirred solution of KSCN (0.11 mol) in dry acetone (50 ml), followed by slow addition of 2-nitroaniline (0.1 mol) in dry acetone (25 ml). The mixture was refluxed for 5–10 min, then poured on ice cooled water, which resulted in crude precipitate. Recrystallization of the precipitate from ethylacetate yielded colourless needles.

### Refinement

The H-atoms were positioned geometrically (C—H = 0.93–0.96 Å, N—H = 0.86 Å) and refined as riding with *U*_iso_(H) = *xU*_eq_(C, N), where *x* = 1.5 for methyl groups and *x* = 1.2 for other H atoms.

### Crystal data

**Table:**

C_9_H_9_N_3_O_3_S	*F*(000) = 496
*M**_r_* = 239.25	*D*_x_ = 1.480 Mg m^−^^3^
Monoclinic, *P*2_1_/*n*	Mo *K*α radiation, λ = 0.71073 Å
Hall symbol: -P 2yn	Cell parameters from 1969 reflections
*a* = 4.1992 (1) Å	θ = 1.9–28.4°
*b* = 11.6081 (3) Å	µ = 0.30 mm^−^^1^
*c* = 22.1035 (6) Å	*T* = 296 K
β = 94.815 (1)°	Needle, colourless
*V* = 1073.63 (5) Å^3^	0.35 × 0.15 × 0.13 mm
*Z* = 4	

### Data collection

**Table:**

Bruker Kappa APEXII CCD diffractometer	2711 independent reflections
Radiation source: fine-focus sealed tube	1969 reflections with *I* > 2σ(*I*)
Graphite monochromator	*R*_int_ = 0.029
Detector resolution: 7.50 pixels mm^-1^	θ_max_ = 28.4°, θ_min_ = 1.9°
ω scans	*h* = −5→5
Absorption correction: multi-scan (*SADABS*; Bruker, 2005)	*k* = −15→15
*T*_min_ = 0.945, *T*_max_ = 0.965	*l* = −29→29
10065 measured reflections	

### Refinement

**Table:**

Refinement on *F*^2^	Primary atom site location: structure-invariant direct methods
Least-squares matrix: full	Secondary atom site location: difference Fourier map
*R*[*F*^2^ > 2σ(*F*^2^)] = 0.039	Hydrogen site location: inferred from neighbouring sites
*wR*(*F*^2^) = 0.105	H-atom parameters constrained
*S* = 1.01	*w* = 1/[σ^2^(*F*_o_^2^) + (0.0428*P*)^2^ + 0.3324*P*] where *P* = (*F*_o_^2^ + 2*F*_c_^2^)/3
2711 reflections	(Δ/σ)_max_ < 0.001
146 parameters	Δρ_max_ = 0.22 e Å^−^^3^
0 restraints	Δρ_min_ = −0.22 e Å^−^^3^

### Special details

**Table:**

Geometry. Bond distances, angles *etc*. have been calculated using the rounded fractional coordinates. All su's are estimated from the variances of the (full) variance-covariance matrix. The cell e.s.d.'s are taken into account in the estimation of distances, angles and torsion angles
Refinement. Refinement of *F*^2^ against ALL reflections. The weighted *R*-factor *wR* and goodness of fit *S* are based on *F*^2^, conventional *R*-factors *R* are based on *F*, with *F* set to zero for negative *F*^2^. The threshold expression of *F*^2^ > σ(*F*^2^) is used only for calculating *R*-factors(gt) *etc*. and is not relevant to the choice of reflections for refinement. *R*-factors based on *F*^2^ are statistically about twice as large as those based on *F*, and *R*- factors based on ALL data will be even larger.

### Fractional atomic coordinates and isotropic or equivalent isotropic displacement parameters (Å^2^)

**Table:**

	*x*	*y*	*z*	*U*_iso_*/*U*_eq_	
S1	1.09141 (12)	0.18209 (4)	0.01646 (2)	0.0470 (2)	
O1	1.0422 (5)	0.23102 (15)	0.24000 (7)	0.0772 (7)	
O2	1.4312 (4)	0.3495 (2)	0.25974 (8)	0.1053 (8)	
O3	0.5502 (4)	0.04876 (12)	0.17207 (6)	0.0676 (5)	
N1	1.1962 (4)	0.31649 (18)	0.22843 (7)	0.0608 (6)	
N2	0.8449 (4)	0.22014 (12)	0.12253 (6)	0.0441 (5)	
N3	0.7962 (3)	0.03702 (12)	0.08415 (6)	0.0405 (4)	
C1	0.9181 (4)	0.33887 (14)	0.12522 (7)	0.0402 (5)	
C2	1.0856 (4)	0.38734 (16)	0.17587 (8)	0.0465 (6)	
C3	1.1536 (5)	0.50364 (19)	0.17923 (11)	0.0654 (8)	
C4	1.0529 (6)	0.57302 (19)	0.13141 (12)	0.0763 (9)	
C5	0.8855 (6)	0.52747 (18)	0.08093 (11)	0.0678 (8)	
C6	0.8168 (5)	0.41127 (16)	0.07781 (9)	0.0537 (7)	
C7	0.9000 (4)	0.14835 (14)	0.07749 (7)	0.0369 (5)	
C8	0.6239 (4)	−0.00808 (16)	0.12923 (8)	0.0449 (6)	
C9	0.5378 (5)	−0.13221 (17)	0.12095 (8)	0.0522 (6)	
H2	0.75624	0.19142	0.15281	0.0529*	
H3	1.26623	0.53434	0.21351	0.0785*	
H3A	0.84485	−0.01078	0.05666	0.0485*	
H4	1.09789	0.65145	0.13307	0.0915*	
H5	0.81794	0.57530	0.04862	0.0814*	
H6	0.70161	0.38146	0.04358	0.0645*	
H9A	0.72667	−0.17858	0.12780	0.0784*	
H9B	0.44403	−0.14442	0.08033	0.0784*	
H9C	0.38729	−0.15343	0.14937	0.0784*	

### Atomic displacement parameters (Å^2^)

**Table:**

	*U*^11^	*U*^22^	*U*^33^	*U*^12^	*U*^13^	*U*^23^
S1	0.0619 (3)	0.0391 (2)	0.0416 (3)	−0.0060 (2)	0.0135 (2)	−0.0040 (2)
O1	0.1231 (15)	0.0614 (10)	0.0455 (9)	0.0175 (10)	−0.0028 (9)	0.0020 (7)
O2	0.0672 (11)	0.177 (2)	0.0673 (11)	0.0072 (12)	−0.0204 (9)	−0.0075 (12)
O3	0.0970 (11)	0.0569 (9)	0.0533 (8)	−0.0117 (8)	0.0325 (8)	−0.0063 (7)
N1	0.0646 (11)	0.0792 (13)	0.0381 (9)	0.0208 (10)	0.0014 (8)	−0.0132 (9)
N2	0.0628 (9)	0.0352 (7)	0.0350 (8)	0.0011 (7)	0.0087 (7)	−0.0019 (6)
N3	0.0512 (8)	0.0335 (7)	0.0371 (7)	0.0002 (6)	0.0064 (6)	−0.0022 (6)
C1	0.0491 (10)	0.0345 (9)	0.0375 (9)	0.0039 (7)	0.0063 (7)	−0.0051 (7)
C2	0.0500 (10)	0.0498 (10)	0.0401 (9)	0.0066 (8)	0.0066 (8)	−0.0085 (8)
C3	0.0704 (14)	0.0558 (13)	0.0700 (14)	−0.0081 (11)	0.0060 (11)	−0.0250 (11)
C4	0.0957 (18)	0.0385 (11)	0.0970 (19)	−0.0106 (11)	0.0224 (15)	−0.0133 (12)
C5	0.0949 (17)	0.0420 (11)	0.0676 (14)	0.0081 (11)	0.0134 (13)	0.0081 (10)
C6	0.0704 (13)	0.0411 (10)	0.0488 (11)	0.0069 (9)	−0.0004 (9)	−0.0004 (8)
C7	0.0411 (9)	0.0341 (8)	0.0345 (8)	0.0033 (7)	−0.0026 (7)	−0.0017 (6)
C8	0.0503 (10)	0.0454 (10)	0.0390 (9)	−0.0017 (8)	0.0033 (8)	0.0036 (8)
C9	0.0594 (12)	0.0500 (11)	0.0470 (10)	−0.0126 (9)	0.0031 (9)	0.0041 (8)

### Geometric parameters (Å, º)

**Table:**

S1—C7	1.6737 (17)	C2—C3	1.381 (3)
O1—N1	1.223 (3)	C3—C4	1.367 (3)
O2—N1	1.219 (2)	C4—C5	1.374 (4)
O3—C8	1.215 (2)	C5—C6	1.380 (3)
N1—C2	1.467 (2)	C8—C9	1.493 (3)
N2—C1	1.412 (2)	C3—H3	0.9300
N2—C7	1.333 (2)	C4—H4	0.9300
N3—C7	1.376 (2)	C5—H5	0.9300
N3—C8	1.383 (2)	C6—H6	0.9300
N2—H2	0.8600	C9—H9A	0.9600
N3—H3A	0.8600	C9—H9B	0.9600
C1—C6	1.382 (3)	C9—H9C	0.9600
C1—C2	1.390 (2)		
			
O1—N1—O2	123.61 (19)	S1—C7—N3	118.95 (12)
O1—N1—C2	118.86 (16)	N2—C7—N3	115.52 (14)
O2—N1—C2	117.45 (19)	O3—C8—C9	123.00 (17)
C1—N2—C7	126.20 (14)	N3—C8—C9	114.40 (15)
C7—N3—C8	128.50 (14)	O3—C8—N3	122.61 (17)
C1—N2—H2	117.00	C2—C3—H3	120.00
C7—N2—H2	117.00	C4—C3—H3	120.00
C7—N3—H3A	116.00	C3—C4—H4	120.00
C8—N3—H3A	116.00	C5—C4—H4	120.00
N2—C1—C2	121.50 (15)	C4—C5—H5	120.00
N2—C1—C6	120.62 (15)	C6—C5—H5	120.00
C2—C1—C6	117.85 (16)	C1—C6—H6	120.00
C1—C2—C3	121.84 (18)	C5—C6—H6	120.00
N1—C2—C1	121.07 (16)	C8—C9—H9A	109.00
N1—C2—C3	117.09 (17)	C8—C9—H9B	109.00
C2—C3—C4	119.0 (2)	C8—C9—H9C	109.00
C3—C4—C5	120.3 (2)	H9A—C9—H9B	109.00
C4—C5—C6	120.5 (2)	H9A—C9—H9C	109.00
C1—C6—C5	120.45 (19)	H9B—C9—H9C	109.00
S1—C7—N2	125.50 (13)		
			
O1—N1—C2—C3	149.7 (2)	C6—C1—C2—N1	178.95 (17)
O1—N1—C2—C1	−30.0 (3)	C6—C1—C2—C3	−0.7 (3)
O2—N1—C2—C1	153.06 (19)	N2—C1—C6—C5	179.21 (19)
O2—N1—C2—C3	−27.3 (3)	C2—C1—C6—C5	0.9 (3)
C7—N2—C1—C2	−129.53 (19)	N2—C1—C2—N1	0.7 (3)
C7—N2—C1—C6	52.2 (3)	N2—C1—C2—C3	−178.95 (17)
C1—N2—C7—S1	4.5 (3)	N1—C2—C3—C4	−179.54 (19)
C1—N2—C7—N3	−177.62 (15)	C1—C2—C3—C4	0.1 (3)
C8—N3—C7—N2	4.8 (2)	C2—C3—C4—C5	0.3 (3)
C7—N3—C8—O3	−2.9 (3)	C3—C4—C5—C6	0.0 (4)
C7—N3—C8—C9	177.33 (16)	C4—C5—C6—C1	−0.6 (3)
C8—N3—C7—S1	−177.15 (13)		

### Hydrogen-bond geometry (Å, º)

**Table:**

*D*—H···*A*	*D*—H	H···*A*	*D*···*A*	*D*—H···*A*
N2—H2···O1	0.86	2.23	2.661 (2)	111
N2—H2···O3	0.86	1.93	2.630 (2)	137
N3—H3*A*···S1^i^	0.86	2.59	3.4371 (14)	168
C9—H9*C*···O2^ii^	0.96	2.51	3.428 (3)	161

Symmetry codes: (i) −*x*+2, −*y*, −*z*; (ii) −*x*+3/2, *y*−1/2, −*z*+1/2.
